# Optoacoustic diagnostic modality: from idea to clinical studies with highly compact laser diode-based systems

**DOI:** 10.1117/1.JBO.22.9.091512

**Published:** 2017-04-25

**Authors:** Rinat O. Esenaliev

**Affiliations:** University of Texas Medical Branch, Laboratory for Optical Sensing and Monitoring, Center for Biomedical Engineering, Department of Neuroscience and Cell Biology, Department of Anesthesiology, Galveston, Texas, United States

**Keywords:** optoacoustic, photoacoustic, monitoring, imaging, cancer detection, thermotherapy, oxygenation

## Abstract

Optoacoustic (photoacoustic) diagnostic modality is a technique that combines high optical contrast and ultrasound spatial resolution. We proposed using the optoacoustic technique for a number of applications, including cancer detection, monitoring of thermotherapy (hyperthermia, coagulation, and freezing), monitoring of cerebral blood oxygenation in patients with traumatic brain injury, neonatal patients, fetuses during late-stage labor, central venous oxygenation monitoring, and total hemoglobin concentration monitoring as well as hematoma detection and characterization. We developed and built optical parametric oscillator-based systems and multiwavelength, fiber-coupled highly compact, laser diode-based systems for optoacoustic imaging, monitoring, and sensing. To provide sufficient output pulse energy, a specially designed fiber-optic system was built and incorporated in ultrasensitive, wideband optoacoustic probes. We performed preclinical and clinical tests of the systems and the optoacoustic probes in backward mode for most of the applications and in forward mode for the breast cancer and cerebral applications. The high pulse energy and repetition rate allowed for rapid data acquisition with high signal-to-noise ratio from cerebral blood vessels, such as the superior sagittal sinus, central veins, and peripheral veins and arteries, as well as from intracranial hematomas. The optoacoustic systems were capable of automatic, real-time, continuous measurements of blood oxygenation in these blood vessels.

## 1 Introduction

Biomedical optics has found a number of important applications in diagnostics and therapy. In part, this is due to high optical absorption contrast of tissue chromophores.^1^ The high endogenous contrast is a major advantage of optical diagnostic techniques compared with other imaging modalities, such as ultrasonography. However, pure optical technologies are not free of limitations associated with limited resolution due to strong light scattering in tissues, in particular, at depths greater than the optical diffusion limit.

Optoacoustic diagnostic modality is based on the generation of ultrasound in tissues by short optical pulses via an absorption-based, thermoelastic mechanism. The amplitude of the generated optoacoustic pressure waves is linearly dependent on the absorption coefficient. Time-resolved detection of the light-induced ultrasound waves yields high (optical) contrast of up to hundreds compared with background absorption and high (ultrasound) resolution of submillimeter or higher that can be used for imaging, sensing, and monitoring in tissues. For more than two decades, we have been working on biomedical optoacoustics. The Organizing Committee of the 24th International Conference Advanced Laser Technologies invited us to present at the conference a review of our optoacoustic works from initial studies to clinical tests with laser-diode optoacoustic systems. This paper represents the invited review of our works on optoacoustic diagnostic applications, development of optoacoustic systems, and tests of the optoacoustic systems in tissue phantoms, tissues *in vitro* and *in vivo*, animal models, and clinical studies.

## 2 Detection of Optoacoustic Signals from Tissues

In the early 1990s, we used optoacoustics to study pulsed laser ablation of tissues and tissue-like media.^2^ Pulsed laser light can ablate tissue with minimal thermal and mechanical damage to adjacent tissues. We quantitatively studied optoacoustic effects induced by laser pulses in tissues. Absolute thermoelastic pressure generated by nanosecond pulses was measured below and above the ablation threshold using specially designed wideband ultrasound transducers with high temporal resolution. The amplitudes and profiles of the optoacoustic pulses generated in atherosclerotic human aorta tissues and aqueous solutions of absorbing dyes were measured at different laser pulse fluences (0.12 to $7.5\;J/{\mathrm{cm}}^{2}$ at 308 nm and 0.75 to $15\;J/{\mathrm{cm}}^{2}$ at 1064 nm). In those studies, we also described informational capabilities of the optoacoustic waves, in particular, for imaging in tissues and for measurement of optical properties of tissues.^2^ Since then we measured thermoelastic and recoil pressure amplitude and duration and studied their effects on tissue phantoms, cells, and tissues.^3^^–^^5^

Short laser pulses (typically, with nanosecond duration) were used for stress-confined irradiation to provide high resolution and contrast in optoacoustic images and generate sufficient optoacoustic pressure amplitude. We used the stress-confined irradiation and time-resolved detection of optoacoustic waves with specially designed wideband acoustic transducers for optoacoustic imaging in turbid tissues as well as for measurement of tissue optical properties and fluence distribution in tissues.^6^^–^^16^

In the mid-1990s, we demonstrated optoacoustic signal detection in tissues at depths well beyond the optical diffusion limit.^7^ We quantitatively studied maximal depth of optoacoustic signal detection, acoustic attenuation of optoacoustic waves, and limit of resolution. Optoacoustic waves induced by Nd:YAG laser pulses at the wavelength of 1064 nm were detected from millimeter-sized liver samples (simulating small tumors with a higher absorption coefficient) surrounded by tissues with a low absorption coefficient (chicken breast muscle). Optoacoustic signals from the tissues with a higher absorption coefficient were measurable at depths up to five times greater than the light penetration depth defined as $1/\mu_{\mathrm{eff}}$, where $\mu_{\mathrm{eff}}$ is the effective attenuation coefficient. The capability of the optoacoustic technique to detect $3\text{-}{\mathrm{mm}}^{3}$ liver samples placed inside 80-mm muscle tissue was shown as well.^7^

## 3 Optoacoustic Imaging

Detection of optically absorbing volumes deeply in tissue is important for a number of diagnostic imaging applications including breast cancer detection. We experimentally and theoretically demonstrated high sensitivity of optoacoustic imaging of small, deeply embedded model tumors at depths of up to several centimeters.^9^ Then we reconstructed optoacoustic images that had a higher contrast compared with ultrasonography and x-ray imaging.^10^

**Fig. 1:**
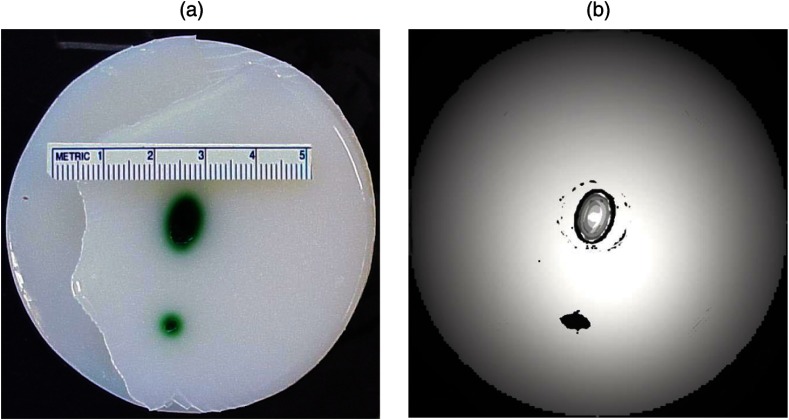
(a) Breast phantom with two model tumors (elliptical and spherical). After data acquisition, the phantom was cut along the optoacoustic image plane. (b) Corresponding optoacoustic image of the breast phantom with the two model tumors.

We developed radial back-projection algorithm for optoacoustic imaging and obtained high-resolution optoacoustic images in turbid tissue phantoms (diameter of up to 10 cm) with small model tumors of different sizes, shapes, and at different depths [Figs. 1(a) and 1(b)].^11^ In that work, optoacoustic signals were detected from the phantoms in the plane of the tumors at multiple angles (180 measurements/angles with an increment of 2 deg for the entire 360 deg scan) to provide images of small and large tumors with high resolution and contrast. Comparison of the optoacoustic, ultrasound, and x-ray images proved that the optoacoustic technique has substantially higher contrast and resolution.^11^ The axial resolution of optoacoustic imaging in the breast phantoms was better than 0.5 mm at depths of up to 4 cm. The results of the study indicated that the optoacoustic imaging may become an important tool for cancer diagnostics, in particular, for detection of tumors <5  mm with submillimeter resolution.

Spatial resolution of optoacoustic images obtained in our works was substantially better than that of optical imaging. However, it may degrade due to attenuation or/and diffraction of optoacoustic waves in tissue. We measured the axial resolution versus acoustic attenuation and diffraction of optoacoustic waves in water with absorbing layers in breast phantoms and tissues.^12^ Absolute values of optoacoustic pressure were measured in the range from 0.02 to 10 mbar with custom-built, calibrated piezoelectric transducers from an absorbing layer or a small absorbing sphere placed in a medium with a lower optical absorption. The distances between the transducer and the absorbing object were measured from recorded optoacoustic pressure profiles and compared with actual distances measured with a micrometer. Our studies indicated that, despite strong acoustic attenuation of high-frequency ultrasonic waves, the axial resolution of laser optoacoustic imaging may be as high as 20  μm for tissue layers located at a 5-mm depth. High axial resolution of 10 to 20  μm was shown for an absorbing layer at a distance of 5 cm in water when the resolution was affected only by diffraction.^12^

## 4 Optoacoustic Monitoring of Thermotherapy

Real-time optoacoustic monitoring of tissue optical properties and speed of sound may provide fast and accurate feedback during thermotherapy with heating or cooling agents. Because amplitude and temporal parameters of optoacoustic waves are dependent on tissue properties, detection and analysis of the optoacoustic waves during thermotherapy in real time may be used to monitor the extent of tissue hyperthermia, coagulation, or freezing with high resolution and contrast.^13^^–^^18^

Accurate temperature mapping with submillimeter spatial resolution may provide precise thermotherapy of abnormal tissues with minimal damage to surrounding normal tissues. Amplitude of optoacoustic pressure waves induced in water increases with temperature mostly due to temperature dependence of the thermal expansion coefficient. We experimentally demonstrated linear dependence of optoacoustic pressure amplitude in tissue phantoms and in tissues (liver and myocardium) from 20°C to 53°C in native tissues and from 60°C to 70°C in coagulated tissues.^13^^,^^14^^,^^16^ In another study, we induced temperature gradients in tissue and tissue-like samples and optoacoustically monitored the temperature distribution.^15^ A multisensor temperature probe inserted in the samples proved that the accuracy and the spatial resolution of optoacoustic temperature monitoring were better than 1°C and 1 mm, respectively.

High-resolution, real-time optoacoustic monitoring of tissue coagulation was performed during conductive heating or interstitial heating by continuous wave (CW) laser light.^15^^,^^16^ Measurement and analysis of optoacoustic signal amplitudes were used to monitor tissue heating and dimensions of coagulation lesions. Moreover, we experimentally demonstrated that tissue hypothermia and freezing can be monitored as well using the optoacoustic technique because both the amplitude and profile of the optoacoustic waves change with temperature during cooling and freezing.^14^

Nanoparticles that strongly absorb light can be used for photothermal therapy of tumors and other abnormal tissues.^19^^–^^21^ Because the optoacoustic technique is sensitive to changes in absorption, one can use it for monitoring of both nanoparticle delivery into tumors and tumor coagulation. We used the optoacoustic technique to monitor in real-time the accumulation of nanoparticles in human tumors of nude mice and the nanoparticle-induced laser thermotherapy of these tumors.^22^

## 5 Optoacoustic Monitoring of Blood Oxygenation and Hemoglobin Concentration

One of the most important optoacoustic applications is oxygenation and hemoglobin concentration imaging, monitoring, and sensing.^23^^,^^24^ Optoacoustic imaging, monitoring, and sensing of these physiologic variables can be used for diagnostics and image-guided therapy in large populations of patients, including those with traumatic brain injury (TBI), circulatory shock, stroke, patients undergoing surgery, anemic patients, neonatal patients, and fetuses during late-stage labor. We proposed and developed a noninvasive, optoacoustic diagnostic platform for measurements of oxygenation, hemoglobin concentration, and other important physiological parameters in tissues and specific blood vessels.^23^^–^^32^ Because hemoglobin is a major chromophore in the near IR spectral range and its absorption depends on oxygenation, optoacoustics is suitable for monitoring of these physiologic variables.

Noninvasive diagnosis of cerebral hypoxia and detection and characterization of hematomas are highly beneficial for patients with TBI, stroke, and other diseases.^33^^,^^34^ Although CT and MRI can diagnose intracranial hematomas, they cannot be used until the patient arrives at a major healthcare facility. Pure optical techniques may suggest the presence of unilateral intracranial hematomas, but they provide limited information on hematoma type, size, and location due to strong light scattering. Therefore, development of optoacoustic diagnosis of intracranial hematoma is necessary to improve outcomes in patients with TBI.

The existing techniques for venous blood oxygenation monitoring are invasive and require catheterization of the internal jugular bulb. Despite strong optical contrast between oxy- and deoxyhemoglobin in the near IR spectral range, pure optical techniques cannot provide cerebral venous blood oxygenation measurements due to strong light scattering in tissues and optical signal interference from other tissues. In contrast to pure optical technologies, the optoacoustic technique allows for localization of blood vessels with high spatial resolution. Our studies demonstrated that optoacoustics can provide accurate measurements of blood oxygenation and other important physiologic variables in veins and arteries^27^^–^^32^^,^^35^^–^^39^ including the superior sagittal sinus (SSS), a large central cerebral vein. The SSS is located right beneath the skull in the midline of the human head and represents a promising site for optoacoustic monitoring of cerebral blood oxygenation.

Newborns (in particular, premature ones) are at increased risk for severe neurological disabilities (including cerebral palsy) associated with cerebral hypoxia. However, no technology is capable of noninvasive, accurate monitoring of cerebral venous oxygenation in neonatal patients. We proposed to use optoacoustics for noninvasive cerebral oxygenation monitoring in neonates by probing intracranial space in the reflection mode through the open anterior or posterior fontanelles as well as through the skull.^38^

**Fig. 2:**
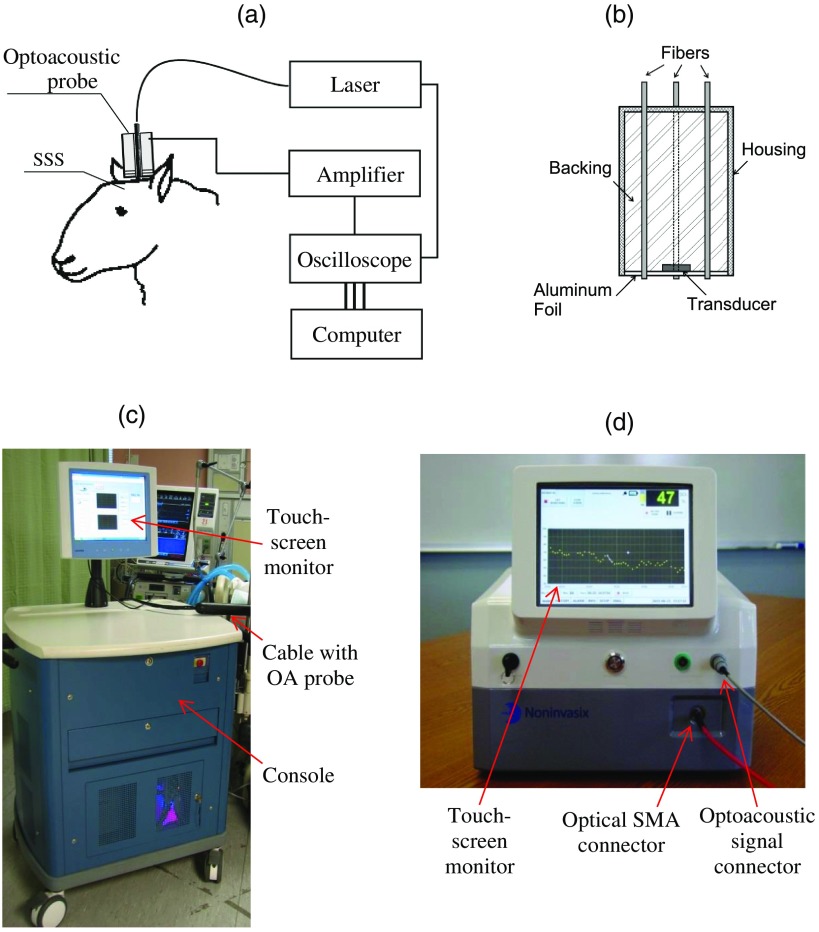
(a) Schematic diagram of the optoacoustic system for animal studies. (b) Optoacoustic probe for reflection and transmission mode measurements. (c) Medical grade, OPO-based optoacoustic system for monitoring of oxygenation and other important physiologic variables. It can be used also for hematoma detection in patients with TBI. (d) Fiber-coupled, multiwavelength, high-power, compact, laser diode-based optoacoustic system for optoacoustic imaging, monitoring, and sensing.

We have developed three types of optoacoustic systems for monitoring, imaging, and sensing: (1) multiwavelength, optical parametric oscillator (OPO)-based systems tunable in a wide spectral range from 680 to 1064 nm for animal studies and fiber-coupled to optoacoustic probes [Figs. 2(a) and 2(b)]; the probes can be used in the reflection and transmission modes for animal and clinical studies; (2) medical grade, multiwavelength, OPO-based fiber-coupled systems [Fig. 2(c)] for clinical studies; and (3) multiwavelength, fiber-coupled, high-power, compact laser diode systems [Fig. 2(d)] for animal and clinical studies. Using these systems, we performed small and large animal and clinical studies on detection and characterization of hematomas and on monitoring cerebral hypoxia. To provide statistically significant data, the studies were performed on 15 rats, 18 sheep, and 12 human subjects.

The clinical protocols for the studies involving human subjects were approved by the Institutional Review Boards of the University of Texas Medical Branch (UTMB) and Baylor College of Medicine. A signed informed consent was obtained for each subject. All animal studies were approved by the Institutional Animals Care and Use Committee of UTMB.

To detect optoacoustic signals with high signal-to-noise ratio, we developed and built ultrasensitive, wideband, optoacoustic probes for monitoring, imaging, and sensing in the reflection and transmission modes.^27^^–^^32^^,^^35^^–^^39^ In the transmission mode, light delivery and detection of optoacoustic signals are performed from opposite hemispheres, whereas in the reflection mode, light delivery and detection of optoacoustic signals are performed from the same hemisphere. We used the reflection mode in all the animal studies as well as in clinical studies in neonates to detect the SSS signals through open anterior or posterior fontanelle.

The transmission mode was used for transcranial detection in adults and neonates of optoacoustic waves induced in the cerebral blood vessels or hematomas. The transducers were placed on the forehead of the subjects, while light was delivered on the opposite side of the head. We developed algorithms and software packages to control the OPO- and laser diode-based systems, provide rapid data acquisition and processing, and measure and display continuously and in real-time oxygenation and other tissue parameters.

We calibrated our systems using tissue phantoms with cylindrical cavities simulating blood vessels and validated oxygenation measurements as described in detail in our publications.^32^^,^^35^^,^^38^ The tissue phantoms were made of tissue-like plastic polyvinyl chloride plastisol with white plastic color added to provide an effective attenuation coefficient close to that for soft tissues in the NIR spectral range. We filled the phantom with fresh heparinized arterial sheep blood and measured the optoacoustic signals. Then, we gradually changed blood oxygenation and performed optoacoustic measurements. The obtained results demonstrated linear dependence of optoacoustic signal amplitudes on oxygenation and were used for calibration and for prediction of oxygenation *in vivo* based on the calibration. We then evaluated the SSS oxygenation accuracy measurements the *in vivo* experiments in sheep. A small craniotomy was made close to the site of optoacoustic measurements. We inserted a catheter into the SSS through this craniotomy to sample blood immediately after every optoacoustic measurement and obtain actual value of cerebral blood oxygenation at the moment with the CO-Oximeter (“gold standard” for oxygenation measurements). Optoacoustic signals were measured using the optoacoustic probe placed over the SSS. The optoacoustically predicted oxygenation correlated well with actual blood oxygenation in sheep SSS ($R^{2}=0.965$ to 0.990). Bland–Altman analysis yielded the mean difference and the standard deviation of 4.8% and 2.8%, respectively.^32^ The large animal studies allow for assessment of the optoacoustic oxygenation monitoring accuracy because blood samples can be taken for reference oxygenation measurements. Small animal studies are not used for this purpose because of the problems associated with catheterization of small blood vessels and severe iatrogenic blood loss relative to the total blood volume in small animals. Another advantage of large animal studies is the similarity of tissue thickness and blood vessel size to that of humans. Using the oxygenation measurement algorithm developed and validated in the sheep studies, we performed optoacoustic monitoring of blood oxygenation in human subjects. It should be noted that catheterizations of the human SSS is not performed due to extremely high risks.

**Fig. 3:**
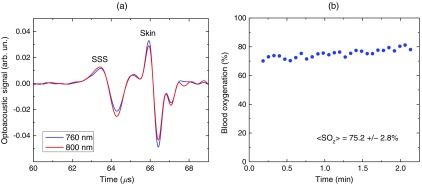
(a) Optoacoustic signal measured in the transmission mode from a premature low-birth-weight newborn (1790 g) using the medical grade optoacoustic system. (b) Continuous, noninvasive optoacoustic monitoring of the SSS blood oxygenation in the newborn.

Figure 3(a) shows optoacoustic signals measured from the head of a premature infant (birth weight: 1790 g) in the transmission mode with the medical grade OPO-based optoacoustic system at 800 nm (red line) and 760 nm (blue line).^38^ The first peak induced in the SSS arrives to the transducer earlier than the peak from the scalp because (in contrast to the reflection mode) the SSS is closer to the transducer than the irradiated skin surface. Because the SSS signal arrives earlier, there is no interfering ringing from the skin signal that can reduce the accuracy of the SSS peak parameters (amplitude and slope) measurements. The multiwavelength measurements allowed for continuous, noninvasive monitoring of the SSS blood oxygenation (${\mathrm{SO}}_{2}$) in the newborn [Fig. 3(b)]. The mean SSS oxygenation ($\left\langle {\mathrm{SO}}_{2}\right\rangle$) and standard deviation were 75% and 3%, respectively. It should be noted that the standard deviation includes precision of system’s measurements, natural variation of ${\mathrm{SO}}_{2}$, and inaccuracy associated with motion artifacts during the monitoring time. Moreover, we used the exponential slope of the SSS signal at 800 nm to measure total hemoglobin concentration (THb=15.7  g/dL which is in good agreement with the actual THb=15.8  g/dL measured invasively using blood sampling). These noninvasive THb measurements can be performed because the optoacoustic signal slope is linearly dependent on blood THb, as experimentally proven in our studies.^24^^,^^26^

**Fig. 4:**
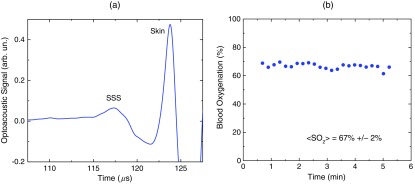
(a) Optoacoustic signals measured in the transmission mode from the SSS of an adult patient with severe TBI. (b) Continuous, noninvasive optoacoustic measurement in the transmission mode of the SSS blood oxygenation in a TBI patient.

Optoacoustic signal measured from the SSS of a patient with TBI at 800 nm is presented in Fig. 4(a).^38^ The first peak (t=117  μs) produced in the SSS arrives to the transducer earlier than the peak from the skin (t=124  μs) because the SSS is closer to the transducer than the skin. The time of flight through the whole adult head (from the scalp in the occipital area to the forehead) is ∼120  μs, which is in good agreement with the adult head size (∼17 to 20 cm). This is because the speed of sound in the brain and other soft tissues is close to 1.5  mm/μs, and the skull occupies only a small fraction of this distance. We continuously measured the SSS blood oxygenation using the optoacoustic signals recorded at different wavelengths in the TBI patients. Figure 4(b) shows that the optoacoustically measured SSS blood oxygenation was $\langle {\mathrm{SO}}_{2}\rangle =67\%\pm 2\%$.

**Fig. 5:**
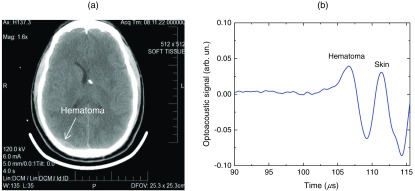
(a) CT scan of a patient with severe TBI. The intracranial hematoma (the lens-shaped structure) is on the right side of the patient head close to the midline. (b) Optoacoustic signal detected from the intracranial hematoma in the transmission mode from the patient with intracranial hematoma.

Optoacoustic signals from patients with TBI were used for intracranial hematoma detection in patients with severe TBI. Figure 5(a) shows a CT scan of a TBI patient’s head with an intracranial hematoma (the lens-shaped structure) on the right side of the patient head close to the midline. Optoacoustic signal detected from this area in the transmission mode has the hematoma peak at 107  μs, while the skin signal arrived to the transducer at 112  μs [Fig. 5(b)].^38^ The total area of the scanning was $5\times 5\;{\mathrm{cm}}^{2}$. The 5-μs time delay is in good agreement with the overlying tissue thickness (∼6  mm for scalp and for skull) because the speed of sound in soft tissue is 1.5  mm/μs, while in the skull, it is twice as high. Compared with the skin peak, the amplitude of the hematoma peak was higher than that of the SSS peak because hematoma had a larger blood volume. We plan to modify the system for detection of hematomas that are located outside the midline area.

Formation of intracranial and extracranial hematomas was monitored in real time in rats with blast-induced TBI.^37^ In that study, we measured optoacoustic signals from the rat brain before and after the blast-induced injury. Real-time analysis of the obtained signals provided simultaneous measurements of oxygenation in the SSS and intracranial hematomas. Gradual increase of the hematoma signal amplitude was monitored continuously and in real time. The obtained results indicate that the optoacoustic technology is capable of simultaneous detection and characterization of hematomas and measurements of cerebral blood oxygenation.

**Fig. 6:**
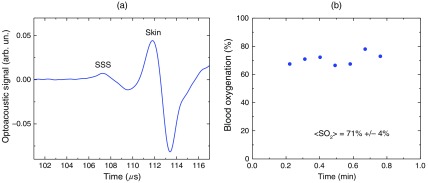
(a) Optoacoustic signals measured in the transmission mode from the adult human SSS using the laser diode-based optoacoustic system. (b) Noninvasive measurement in the transmission mode of the adult human SSS blood oxygenation using the laser diode-based optoacoustic system.

We performed similar studies using the laser diode optoacoustic system.^38^ Optoacoustic signal measured from the adult SSS with the laser diode system [Fig. 6(a)] has the SSS peak at t=107  μs and the skin peak at t=112  μs. The SSS signal had high signal-to-noise ratio due to sufficient pulse energy (0.13 mJ), sensitive detection, and fast optoacoustic signal averaging at the high (1 kHz) pulse repetition rate of the laser diode system that allowed for SSS blood oxygenation measurement with a standard deviation of 4% [Fig. 6(b)]. The fast signal averaging reduces influence of motion artifacts that are more pronounced with slow averaging at the 20-Hz pulse repletion rate of the OPO systems.

The data obtained in neonates and adults indicate that both the OPO- and laser diode-based optoacoustic systems can provide oxygenation measurements from cerebral blood vessels and detection of intracranial hematomas. These results suggest that the optoacoustic technology may be applicable to brain imaging, tomography, and mapping in neonates, children, and adults. We plan to further test the systems’ performance (accuracy, etc.) in clinical studies.

Biomedical optoacoustic field has grown tremendously for the last decade. Recent reviews and publications (and references therein)^40^^–^^51^ discuss remarkable progress in optoacoustic/photoacoustic microscopy, cancer applications, functional and structural imaging in small animals, dual modality optoacoustic/ultrasound imaging, optoacoustic instrumentation, imaging algorithms development, contrast agents development, and other applications. Integration of optoacoustic and ultrasound systems provides optical contrast (functional) and structural (anatomical) information. Laser diode optoacoustic systems without fiber coupling integrated in ultrasound imaging systems were developed for imaging applications in skin and joints.^52^ Although we developed and tested pulsed laser diode systems in our studies, modulated CW laser diodes can be used for optoacoustic imaging, monitoring, and sensing. For instance, a promising, laser diode-based photoacoustic radar imager was proposed and successfully used for blood oxygenation measurements.^53^ Further development of the pulsed and CW laser diode systems will facilitate clinical use of the optoacoustic diagnostic platform.

## 6 Conclusion

We proposed using optoacoustic imaging, monitoring, and sensing for a number of applications and tested the optoacoustic diagnostic platform in tissues, tissue phantoms, animals, and human subjects. Based on the results of these studies, we developed optoacoustic systems that can be used for imaging, monitoring, and sensing. The compact, laser diode-based optoacoustic systems may find wide applications in medical diagnostics and in neuroscience research. The obtained data suggest that this technology may be applicable to large populations of patients (from neonates to adults). The developed optoacoustic systems can be used for single measurement and continuous measurement and monitoring, as well as for 2-D, 3-D, and 4-D imaging of tissues or specific blood vessels. We plan to further develop these systems for clinical applications.
